# What are the strengths and limitations to utilising creative methods in public and patient involvement in health and social care research? A qualitative systematic review

**DOI:** 10.1186/s40900-024-00580-4

**Published:** 2024-05-13

**Authors:** Olivia R. Phillips, Cerian Harries, Jo Leonardi-Bee, Holly Knight, Lauren B. Sherar, Veronica Varela-Mato, Joanne R. Morling

**Affiliations:** 1 Nottingham Centre for Public Health and Epidemiology, Lifespan and Population Health, School of Medicine, University of Nottingham, Clinical Sciences Building, City Hospital Campus, Hucknall Road, Nottingham, NG5 1PB UK; 2 National Institute for Health and Care Research (NIHR) PHIRST-LIGHT, Nottingham, UK; 3 School of Sport, Exercise and Health Sciences, Loughborough University, Epinal Way, Loughborough, Leicestershire, LE11 3TU UK; 4 Nottingham Centre for Evidence Based Healthcare, School of Medicine, University of Nottingham, Nottingham, UK; 5 NIHR Nottingham Biomedical Research Centre (BRC), Nottingham University Hospitals NHS Trust, University of Nottingham, Nottingham, NG7 2UH UK

**Keywords:** Public and patient involvement, PPI, Creative PPI, Qualitative systematic review

## Abstract

**Background:**

There is increasing interest in using patient and public involvement (PPI) in research to improve the quality of healthcare. Ordinarily, traditional methods have been used such as interviews or focus groups. However, these methods tend to engage a similar demographic of people. Thus, creative methods are being developed to involve patients for whom traditional methods are inaccessible or non-engaging.

**Objective:**

To determine the strengths and limitations to using creative PPI methods in health and social care research.

**Method:**

Electronic searches were conducted over five databases on 14th April 2023 (Web of Science, PubMed, ASSIA, CINAHL, Cochrane Library). Studies that involved traditional, non-creative PPI methods were excluded. Creative PPI methods were used to engage with people as research advisors, rather than study participants. Only primary data published in English from 2009 were accepted. Title, abstract and full text screening was undertaken by two independent reviewers before inductive thematic analysis was used to generate themes.

**Results:**

Twelve papers met the inclusion criteria. The creative methods used included songs, poems, drawings, photograph elicitation, drama performance, visualisations, social media, photography, prototype development, cultural animation, card sorting and persona development. Analysis identified four limitations and five strengths to the creative approaches. Limitations included the time and resource intensive nature of creative PPI, the lack of generalisation to wider populations and ethical issues. External factors, such as the lack of infrastructure to support creative PPI, also affected their implementation. Strengths included the disruption of power hierarchies and the creation of a safe space for people to express mundane or “taboo” topics. Creative methods are also engaging, inclusive of people who struggle to participate in traditional PPI and can also be cost and time efficient.

**Conclusion:**

‘Creative PPI’ is an umbrella term encapsulating many different methods of engagement and there are strengths and limitations to each. The choice of which should be determined by the aims and requirements of the research, as well as the characteristics of the PPI group and practical limitations. Creative PPI can be advantageous over more traditional methods, however a hybrid approach could be considered to reap the benefits of both. Creative PPI methods are not widely used; however, this could change over time as PPI becomes embedded even more into research.

**Supplementary Information:**

The online version contains supplementary material available at 10.1186/s40900-024-00580-4.

## Abstract (plain language summary)

It is important that patients and public are included in the research process from initial brainstorming, through design to delivery. This is known as public and patient involvement (PPI). Their input means that research closely aligns with their wants and needs. Traditionally to get this input, interviews and group discussions are held, but this can exclude people who find these activities non-engaging or inaccessible, for example those with language challenges, learning disabilities or memory issues. Creative methods of PPI can overcome this. This is a broad term describing different (non-traditional) ways of engaging patients and public in research, such as through the use or art, animation or performance. This review investigated the reasons why creative approaches to PPI could be difficult (limitations) or helpful (strengths) in health and social care research. After searching 5 online databases, 12 studies were included in the review. PPI groups included adults, children and people with language and memory impairments. Creative methods included songs, poems, drawings, the use of photos and drama, visualisations, Facebook, creating prototypes, personas and card sorting. Limitations included the time, cost and effort associated with creative methods, the lack of application to other populations, ethical issues and buy-in from the wider research community. Strengths included the feeling of equality between academics and the public, creation of a safe space for people to express themselves, inclusivity, and that creative PPI can be cost and time efficient. Overall, this review suggests that creative PPI is worthwhile, however each method has its own strengths and limitations and the choice of which will depend on the research project, PPI group characteristics and other practical limitations, such as time and financial constraints.

**Supplementary Information:**

The online version contains supplementary material available at 10.1186/s40900-024-00580-4.

## Introduction

Patient and public involvement (PPI) is the term used to describe the partnership between patients (including caregivers, potential patients, healthcare users etc.) or the public (a community member with no known interest in the topic) with researchers. It describes research that is done “‘with’ or ‘by’ the public, rather than ‘to,’ ‘about’ or ‘for’ them” [1]. In 2009, it became a legislative requirement for certain health and social care organisations to include patients, families, carers and communities in not only the planning of health and social care services, but the commissioning, delivery and evaluation of them too [2]. For example, funding applications for the National Institute of Health and Care Research (NIHR), a UK funding body, mandates a demonstration of how researchers plan to include patients/service users, the public and carers at each stage of the project [3]. However, this should not simply be a tokenistic, tick-box exercise. PPI should help formulate initial ideas and should be an instrumental, continuous part of the research process. Input from PPI can provide unique insights not yet considered and can ensure that research and health services are closely aligned to the needs and requirements of service users PPI also generally makes research more relevant with clearer outcomes and impacts [4]. Although this review refers to both patients and the public using the umbrella term ‘PPI’, it is important to acknowledge that these are two different groups with different motivations, needs and interests when it comes to health research and service delivery [5].

Despite continuing recognition of the need of PPI to improve quality of healthcare, researchers have also recognised that there is no ‘one size fits all’ method for involving patients [4]. Traditionally, PPI methods invite people to take part in interviews or focus groups to facilitate discussion, or surveys and questionnaires. However, these can sometimes be inaccessible or non-engaging for certain populations. For example, someone with communication difficulties may find it difficult to engage in focus groups or interviews. If individuals lack the appropriate skills to interact in these types of scenarios, they cannot take advantage of the participation opportunities it can provide [6]. Creative methods, however, aim to resolve these issues. These are a relatively new concept whereby researchers use creative methods (e.g., artwork, animations, Lego), to make PPI more accessible and engaging for those whose voices would otherwise go unheard. They ensure that all populations can engage in research, regardless of their background or skills. Seminal work has previously been conducted in this area, which brought to light the use of creative methodologies in research. Leavy (2008) [7] discussed how traditional interviews had limits on what could be expressed due to their sterile, jargon-filled and formulaic structure, read by only a few specialised academics. It was this that called for more creative approaches, which included narrative enquiry, fiction-based research, poetry, music, dance, art, theatre, film and visual art. These practices, which can be used in any stage of the research cycle, supported greater empathy, self-reflection and longer-lasting learning experiences compared to interviews [7]. They also pushed traditional academic boundaries, which made the research accessible not only to researchers, but the public too. Leavy explains that there are similarities between arts-based approaches and scientific approaches: both attempts to investigate what it means to be human through exploration, and used together, these complimentary approaches can progress our understanding of the human experience [7]. Further, it is important to acknowledge the parallels and nuances between creative and inclusive methods of PPI. Although creative methods aim to be inclusive (this should underlie any PPI activity, whether creative or not), they do not incorporate *all* types of accessible, inclusive methodologies e.g., using sign language for people with hearing impairments or audio recordings for people who cannot read. Given that there was not enough scope to include an evaluation of all possible inclusive methodologies, this review will focus on *creative* methods of PPI only.

We aimed to conduct a qualitative systematic review to highlight the strengths of creative PPI in health and social care research, as well as the limitations, which might act as a barrier to their implementation. A qualitative systematic review “brings together research on a topic, systematically searching for research evidence from primary qualitative studies and drawing the findings together” [8]. This review can then advise researchers of the best practices when designing PPI.

## Methods

### Public involvement

The PHIRST-LIGHT Public Advisory Group (PAG) consists of a team of experienced public contributors with a diverse range of characteristics from across the UK. The PAG was involved in the initial question setting and study design for this review.

### Search strategy

For the purpose of this review, the JBI approach for conducting qualitative systematic reviews was followed [9]. The search terms were (“creativ*” OR “innovat*” OR “authentic” OR “original” OR “inclu*”) AND (“public and patient involvement” OR “patient and public involvement” OR “public and patient involvement and engagement” OR “patient and public involvement and engagement” OR “PPI” OR “PPIE” OR “co-produc*” OR “co-creat*” OR “co-design*” OR “cooperat*” OR “co-operat*”). This search string was modified according to the requirements of each database. Papers were filtered by title, abstract and keywords (see Additional file 1 for search strings). The databases searched included Web of Science (WoS), PubMed, ASSIA and CINAHL. The Cochrane Library was also searched to identify relevant reviews which could lead to the identification of primary research. The search was conducted on 14/04/23. As our aim was to report on the use of creative PPI in research, rather than more generic public engagement, we used electronic databases of scholarly peer-reviewed literature, which represent a wide range of recognised databases. These identified studies published in general international journals (WoS, PubMed), those in social sciences journals (ASSIA), those in nursing and allied health journals (CINAHL), and trials of interventions (Cochrane Library).

### Inclusion criteria

Only full-text, English language, primary research papers from 2009 to 2023 were included. This was the chosen timeframe as in 2009 the Health and Social Reform Act made it mandatory for certain Health and Social Care organisations to involve the public and patients in planning, delivering, and evaluating services [2]. Only creative methods of PPI were accepted, rather than traditional methods, such as interviews or focus groups. For the purposes of this paper, creative PPI included creative art or arts-based approaches (e.g., e.g. stories, songs, drama, drawing, painting, poetry, photography) to enhance engagement. Titles were related to health and social care and the creative PPI was used to engage with people as research advisors, not as study participants. Meta-analyses, conference abstracts, book chapters, commentaries and reviews were excluded. There were no limits concerning study location or the demographic characteristics of the PPI groups. Only qualitative data were accepted.

### Quality appraisal

Quality appraisal using the Critical Appraisal Skills Programme (CASP) checklist [10] was conducted by the primary authors (ORP and CH). This was done independently, and discrepancies were discussed and resolved. If a consensus could not be reached, a third independent reviewer was consulted (JRM). The full list of quality appraisal questions can be found in Additional file 2.

### Data extraction

ORP extracted the study characteristics and a subset of these were checked by CH. Discrepancies were discussed and amendments made. Extracted data included author, title, location, year of publication, year study was carried out, research question/aim, creative methods used, number of participants, mean age, gender, ethnicity of participants, setting, limitations and strengths of creative PPI and main findings.

### Data analysis

The included studies were analysed using inductive thematic analysis [11], where themes were determined by the data. The familiarisation stage took place during full-text reading of the included articles. Anything identified as a strength or limitation to creative PPI methods was extracted verbatim as an initial code and inputted into the data extraction Excel sheet. Similar codes were sorted into broader themes, either under ‘strengths’ or ‘limitations’ and reviewed. Themes were then assigned a name according to the codes.

## Results

**Fig. 1:**
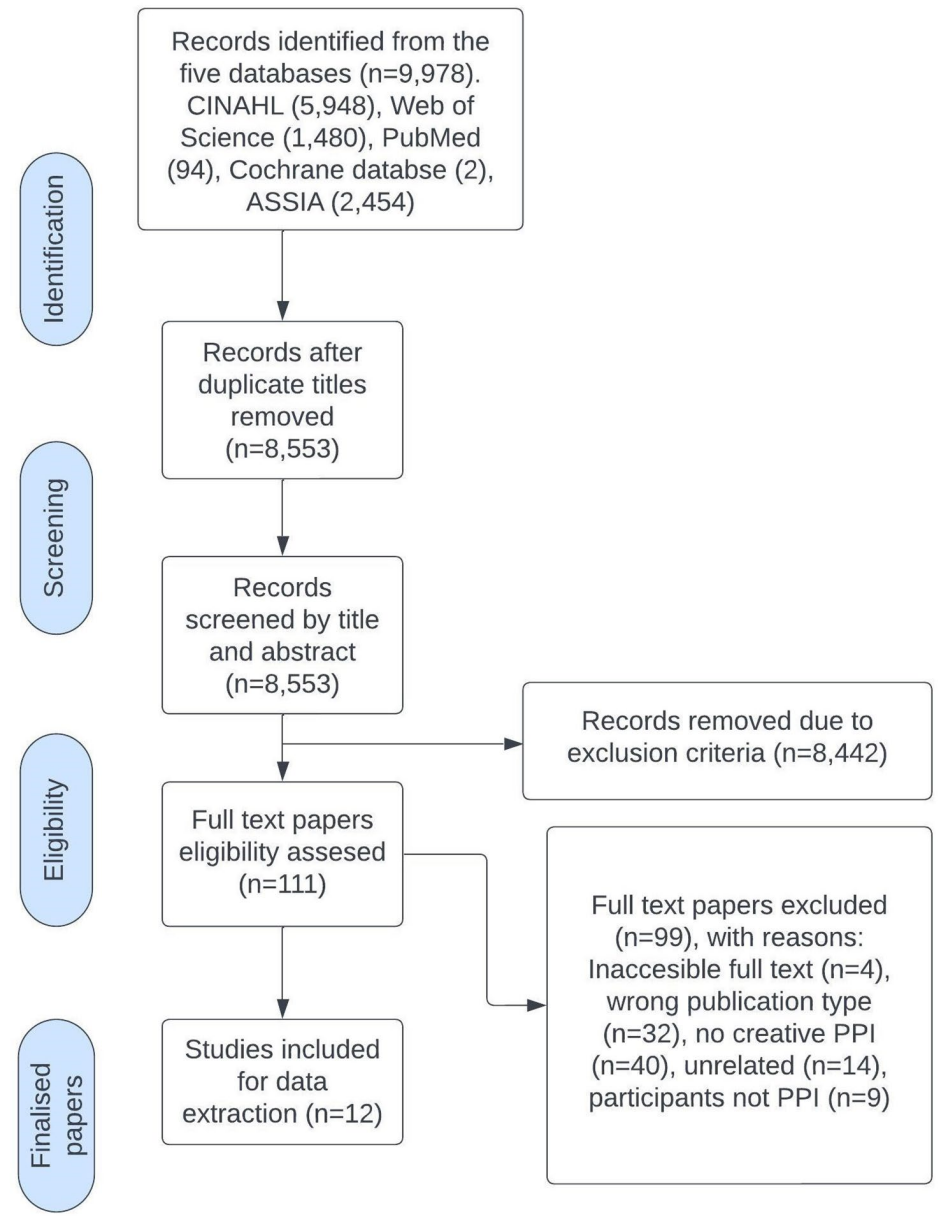
PRISMA flowchart of the study selection process

The search yielded 9978 titles across the 5 databases: Web of Science (1480 results), PubMed (94 results), ASSIA (2454 results), CINAHL (5948 results) and Cochrane Library (2 results), resulting in 8553 different studies after deduplication. ORP and CH independently screened their titles and abstracts, excluding those that did not meet the criteria. After assessment, 12 studies were included (see Fig. 1).

### Study characteristics

The included studies were published between 2018 and 2022. Seven were conducted in the UK [12, 14, 15, 17–19, 23], two in Canada [21, 22], one in Australia [13], one in Norway [16] and one in Ireland [20]. The PPI activities occurred across various settings, including a school [12], social club [12], hospital [17], university [22], theatre [19], hotel [20], or online [15, 21], however this information was omitted in 5 studies [13, 14, 16, 18, 23]. The number of people attending the PPI sessions varied, ranging from 6 to 289, however the majority (ten studies) had less than 70 participants [13, 14, 16–23]. Seven studies did not provide information on the age or gender of the PPI groups. Of those that did, ages ranged from 8 to 76 and were mostly female. The ethnicities of the PPI group members were also rarely recorded (see Additional file 3 for data extraction table).

### Types of creative methods

The type of creative methods used to engage the PPI groups were varied. These included songs, poems, drawings, photograph elicitation, drama performance, visualisations, Facebook, photography, prototype development, cultural animation, card sorting and creating personas (see Table 1). These were sometimes accompanied by traditional methods of PPI such as interviews and focus group discussions.

**Table 1:** Included studies

Author	Location	Year of study/publication	Age, yrs	Gender	Ethnicity	Setting	Creative methods
Byrne et al. [12]	Wales, UK	NS/2018	NS, but included ‘school children’, ‘older people’ and ‘young people’.	NS	NS	Social club, school	Poems, song/music video, drawings, photograph elicitation, theatre performance (alongside semi-structured interviews, focus groups and observation).
Cook et al. [13]	Australia	NS/2021	15–25	“Mixed sex”	NS	NS	Life Happens educational resource / hypothetical scenarios / condom mapping.
Craven et al. [14]	UK	2017/2019	NS	NS	NS	NS	Arts-based visualisations, sticky notes, flipchart paper, coloured pens
Fedorowicz et al. [15]	UK	2016/2022	18–65+	86% female, 40% male	NS	Facebook	Closed Facebook group
Galler et al. [16]	Norway	2021/2022	9–12	11 girls, 10 boys	NS	NS	Newspaper article brainstorming, Padlet Backpack, online food blog, photography.
Grindell et al. [17]	UK	NS/2020	NS	NS	NS	Hospital and teleconferencing	Prototype development, personas, worksheets
Kearns et al. [18]	UK	2019/2019	Mean age 60.7	5 males, 1 female	NS	NS	Visual analogue scales, ranking tasks, photo-diaries, use of prototypes.
Kelemen et al. [19]	Stoke on Trent, England	2013/2018	Age range 25–75 (80% - over the age of 60)	80% women	NS	Theatre	Cultural animation (CA) accompanied by interviews.
Keogh et al. [20]	Ireland	NS/2021	NS	5 male 5 female	NS	Hotel	Card sorting and priority setting
Micsinszki et al. [21]	Canada	2020/2021	NS	NS	NS	Zoom	Google Jamboard™ and boundary objects
Valaitis et al. [22]	Canada	2015/2019	NS	NS	NS	University research centre	Persona scenarios
Webber et al. [23]	Sheffield, England	NS/2022	“adults”	NS	NS	NS	Sketching, collaging, worksheets, personas, boundary objects, mood boards

**NS** not specified;

### Quality appraisal

The 12 included studies were all deemed to be of good methodological quality, with scores ranging from 6/10 to 10/10 with the CASP critical appraisal tool [10] (Table 2).

**Table 2:** Quality appraisal of the included studies

	Q1	Q2	Q3	Q4	Q5	Q6	Q7	Q8	Q9	Q10	Total/10
Byrne et al. 2018 [12]	Y	Y	Y	N	Y	N	N	N	Y	Y	6
Cook et al. 2022 [13]	Y	Y	Y	Y	Y	N	N	Y	Y	Y	8
Craven et al. 2019 [14]	Y	Y	Y	N	Y	N	Y	Y	Y	Y	8
Fedorowicz et al. 2022 [15]	Y	Y	Y	Y	Y	N	N	Y	Y	Y	8
Galler et al. 2022 [16]	Y	Y	Y	Y	Y	N	Y	Y	Y	Y	9
Grindell et al. 2020 [17]	Y	Y	Y	N	Y	N	Y	Y	Y	Y	8
Kearns et al. 2020 [18]	Y	Y	Y	Y	Y	Y	Y	Y	Y	Y	10
Kelemen et al. 2018 [19]	Y	Y	Y	N	Y	Y	Y	Y	Y	Y	9
Keogh et al. 2021 [20]	Y	Y	Y	Y	Y	N	Y	Y	N	Y	8
Micsinszki et al. 2022 [21]	N	Y	Y	Y	Y	N	N	Y	Y	Y	7
Valaitis et al. 2019 [22]	Y	Y	Y	Y	Y	N	Y	Y	Y	Y	9
Webber et al. 2022 [23]	Y	Y	Y	Y	Y	N	Y	Y	Y	Y	9

### Thematic analysis

**Fig. 2:**
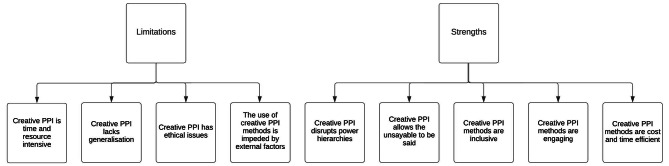
Theme map of strengths and limitations

Analysis identified four limitations and five strengths to creative PPI (see Fig. 2). Limitations included the time and resource intensity of creative PPI methods, its lack of generalisation, ethical issues and external factors. Strengths included the disruption of power hierarchies, the engaging and inclusive nature of the methods and their long-term cost and time efficiency. Creative PPI methods also allowed mundane and “taboo” topics to be discussed within a safe space.

### Limitations of creative PPI

#### Creative PPI methods are time and resource intensive

The time and resource intensive nature of creative PPI methods is a limitation, most notably for the persona-scenario methodology. Valaitis et al. [22] used 14 persona-scenario workshops with 70 participants to co-design a healthcare intervention, which aimed to promote optimal aging in Canada. Using the persona method, pairs composed of patients, healthcare providers, community service providers and volunteers developed a fictional character which they believed represented an ‘end-user’ of the healthcare intervention. Due to the depth and richness of the data produced the authors reported that it was time consuming to analyse. Further, they commented that the amount of information was difficult to disseminate to scientific leads and present at team meetings. Additionally, to ensure the production of high-quality data, to probe for details and lead group discussion there was a need for highly skilled facilitators. The resource intensive nature of the creative co-production was also noted in a study using the persona scenario and creative worksheets to develop a prototype decision support tool for individuals with malignant pleural effusion [17]. With approximately 50 people, this was also likely to yield a high volume of data to consider.

To prepare materials for populations who cannot engage in traditional methods of PPI was also timely. Kearns et al. [18] developed a feedback questionnaire for people with aphasia to evaluate ICT-delivered rehabilitation. To ensure people could participate effectively, the resources used during the workshops, such as PowerPoints, online images and photographs, had to be aphasia-accessible, which was labour and time intensive. The author warned that this time commitment should not be underestimated.

There are further practical limitations to implementing creative PPI, such as the costs of materials for activities as well as hiring a space for workshops. For example, the included studies in this review utilised pens, paper, worksheets, laptops, arts and craft supplies and magazines and took place in venues such as universities, a social club, and a hotel. Further, although not limited to creative PPI methods exclusively but rather most studies involving the public, a financial incentive was often offered for participation, as well as food, parking, transport and accommodation [21, 22].

#### Creative PPI lacks generalisation

Another barrier to the use of creative PPI methods in health and social care research was the individual nature of its output. Those who participate, usually small in number, produce unique creative outputs specific to their own experiences, opinions and location. Craven et al. [13], used arts-based visualisations to develop a toolbox for adults with mental health difficulties. They commented, “such an approach might still not be worthwhile”, as the visualisations were individualised and highly personal. This indicates that the output may fail to meet the needs of its end-users. Further, these creative PPI groups were based in certain geographical regions such as Stoke-on-Trent [19] Sheffield [23], South Wales [12] or Ireland [20], which limits the extent the findings can be applied to wider populations, even within the same area due to individual nuances. Further, the study by Galler et al. [16], is specific to the Norwegian context and even then, maybe only a sub-group of the Norwegian population as the sample used was of higher socioeconomic status.

However, Grindell et al. [17], who used persona scenarios, creative worksheets and prototype development, pointed out that the purpose of this type of research is to improve a certain place, rather than apply findings across other populations and locations. Individualised output may, therefore, only be a limitation to research wanting to conduct PPI on a large scale.

If, however, greater generalisation within PPI is deemed necessary, then social media may offer a resolution. Fedorowicz et al. [15], used Facebook to gain feedback from the public on the use of video-recording methodology for an upcoming project. This had the benefit of including a more diverse range of people (289 people joined the closed group), who were spread geographically around the UK, as well as seven people from overseas.

#### Creative PPI has ethical issues

As with other research, ethical issues must be taken into consideration. Due to the nature of creative approaches, as well as the personal effort put into them, people often want to be recognised for their work. However, this compromises principles so heavily instilled in research such as anonymity and confidentiality. With the aim of exploring issues related to health and well-being in a town in South Wales, Byrne et al. [12], asked year 4/5 and year 10 pupils to create poems, songs, drawings and photographs. Community members also created a performance, mainly of monologues, to explore how poverty and inequalities are dealt with. Byrne noted the risks of these arts-based approaches, that being the possibility of over-disclosure and consequent emotional distress, as well as people’s desire to be named for their work. On one hand, the anonymity reduces the sense of ownership of the output as it does not portray a particular individual’s lived experience anymore. On the other hand, however, it could promote a more honest account of lived experience. Supporting this, Webber et al. [23], who used the persona method to co-design a back pain educational resource prototype, claimed that the anonymity provided by this creative technique allowed individuals to externalise and anonymise their own personal experience, thus creating a more authentic and genuine resource for future users. This implies that anonymity can be both a limitation and strength here.

#### The use of creative PPI methods is impeded by external factors

Despite the above limitations influencing the implementation of creative PPI techniques, perhaps the most influential is that creative methodologies are simply not mainstream [19]. This could be linked to the issues above, like time and resource intensity, generalisation and ethical issues but it is also likely to involve more systemic factors within the research community. Micsinszki et al. [21], who co-designed a hub for the health and well-being of vulnerable populations, commented that there is insufficient infrastructure to conduct meaningful co-design as well as a dominant medical model. Through a more holistic lens, there are “sociopolitical environments that privilege individualism over collectivism, self-sufficiency over collaboration, and scientific expertise over other ways of knowing based on lived experience” [21]. This, it could be suggested, renders creative co-design methodologies, which are based on the foundations of collectivism, collaboration and imagination an invalid technique in the research field, which is heavily dominated by more scientific methods offering reproducibility, objectivity and reliability.

Although we acknowledge that creative PPI techniques are not always appropriate, it may be that their main limitation is the lack of awareness of these methods or lack of willingness to use them. Further, there is always the risk that PPI, despite being a mandatory part of research, is used in a tokenistic or tick-box fashion [20], without considering the contribution that meaningful PPI could make to enhancing the research. It may be that PPI, let alone creative PPI, is not at the forefront of researchers’ minds when planning research.

### Strengths of creative PPI

#### Creative PPI disrupts power hierarchies

One of the main strengths of creative PPI techniques, cited most frequently in the included literature, was that they disrupt traditional power hierarchies [12, 13, 17, 19, 23]. For example, the use of theatre performance blurred the lines between professional and lay roles between the community and policy makers [12]. Individuals created a monologue to portray how poverty and inequality impact daily life and presented this to representatives of the National Assembly of Wales, Welsh Government, the Local Authority, Arts Council and Westminster. Byrne et al. [12], states how this medium allowed the community to engage with the people who make decisions about their lives in an environment of respect and understanding, where the hierarchies are not as visible as in other settings, e.g., political surgeries. Creative PPI methods have also removed traditional power hierarchies between researchers and adolescents. Cook et al. [13], used arts-based approaches to explore adolescents’ ideas about the “perfect” condom. They utilised the “Life Happens” resource, where adolescents drew and then decorated a person with their thoughts about sexual relationships, not too dissimilar from the persona-scenario method. This was then combined with hypothetical scenarios about sexuality. A condom-mapping exercise was then implemented, where groups shared the characteristics that make a condom “perfect” on large pieces of paper. Cook et al. [13], noted that usually power imbalances make it difficult to elicit information from adolescents, however these power imbalances were reduced due to the use of creative co-design techniques.

The same reduction in power hierarchies was noted by Grindell et al. [17], who used the person-scenario method and creative worksheets with individuals with malignant pleural effusion. This was with the aim of developing a prototype of a decision support tool for patients to help with treatment options. Although this process involved a variety of stakeholders, such as patients, carers and healthcare professionals, creative co-design was cited as a mechanism that worked to reduce power imbalances – a limitation of more traditional methods of research. Creative co-design blurred boundaries between end-users and clinical staff and enabled the sharing of ideas from multiple, valuable perspectives, meaning the prototype was able to suit user needs whilst addressing clinical problems.

Similarly, a specific creative method named cultural animation was also cited to dissolve hierarchies and encourage equal contributions from participants. Within this arts-based approach, Keleman et al. [19], explored the concept of “good health” with individuals from Stoke-on Trent. Members of the group created art installations using ribbons, buttons, cardboard and straws to depict their idea of a “healthy community”, which was accompanied by a poem. They also created a 3D Facebook page and produced another poem or song addressing the government to communicate their version of a “picture of health”. Public participants said that they found the process empowering, honest, democratic, valuable and practical.

This dissolving of hierarchies and levelling of power is beneficial as it increases the sense of ownership experienced by the creators/producers of the output [12, 17, 23]. This is advantageous as it has been suggested to improve its quality [23].

#### Creative PPI allows the unsayable to be said

Creative PPI fosters a safe space for mundane or taboo topics to be shared, which may be difficult to communicate using traditional methods of PPI. For example, the hypothetical nature of condom mapping and persona-scenarios meant that adolescents could discuss a personal topic without fear of discrimination, judgement or personal disclosure [13]. The safe space allowed a greater volume of ideas to be generated amongst peers where they might not have otherwise. Similarly, Webber et al. [23], , who used the persona method to co-design the prototype back pain educational resource, also noted how this method creates anonymity whilst allowing people the opportunity to externalise personal experiences, thoughts and feelings. Other creative methods were also used, such as drawing, collaging, role play and creating mood boards. A cardboard cube (labelled a “magic box”) was used to symbolise a physical representation of their final prototype. These creative methods levelled the playing field and made personal experiences accessible in a safe, open environment that fostered trust, as well as understanding from the researchers.

It is not only sensitive subjects that were made easier to articulate through creative PPI. The communication of mundane everyday experiences were also facilitated, which were deemed typically ‘unsayable’. This was specifically given in the context of describing intangible aspects of everyday health and wellbeing [11]. Graphic designers can also be used to visually represent the outputs of creative PPI. These captured the movement and fluidity of people and well as the relationships between them - things that cannot be spoken but can be depicted [21].

#### Creative PPI methods are inclusive

Another strength of creative PPI was that it is inclusive and accessible [17, 19, 21]. The safe space it fosters, as well as the dismantling of hierarchies, welcomed people from a diverse range of backgrounds and provided equal opportunities [21], especially for those with communication and memory difficulties who might be otherwise excluded from PPI. Kelemen et al. [19], who used creative methods to explore health and well-being in Stoke-on-Trent, discussed how people from different backgrounds came together and connected, discussed and reached a consensus over a topic which evoked strong emotions, that they all have in common. Individuals said that the techniques used “sets people to open up as they are not overwhelmed by words”. Similarly, creative activities, such as the persona method, have been stated to allow people to express themselves in an inclusive environment using a common language. Kearns et al. [18], who used aphasia-accessible material to develop a questionnaire with aphasic individuals, described how they felt comfortable in contributing to workshops (although this material was time-consuming to make, see *‘Limitations of creative PPI’*).

Despite the general inclusivity of creative PPI, it can also be exclusive, particularly if online mediums are used. Fedorowicz et al. [15], used Facebook to create a PPI group, and although this may rectify previous drawbacks about lack of generalisation of creative methods (as Facebook can reach a greater number of people, globally), it excluded those who are not digitally active or have limited internet access or knowledge of technology. Online methods have other issues too. Maintaining the online group was cited as challenging and the volume of responses required researchers to interact outside of their working hours. Despite this, online methods like Facebook are very accessible for people who are physically disabled.

#### Creative PPI methods are engaging

The process of creative PPI is typically more engaging and produces more colourful data than traditional methods [13]. Individuals are permitted and encouraged to explore a creative self [19], which can lead to the exploration of new ideas and an overall increased enjoyment of the process. This increased engagement is particularly beneficial for younger PPI groups. For example, to involve children in the development of health food products, Galler et al. [16] asked 9-12-year-olds to take photos of their food and present it to other children in a “show and tell” fashion. They then created a newspaper article describing a new healthy snack. In this creative focus group, children were given lab coats to further their identity as inventors. Galler et al. [16], notes that the methods were highly engaging and facilitated teamwork and group learning. This collaborative nature of problem-solving was also observed in adults who used personas and creative worksheets to develop the resource for lower back pain [23]. Dementia patients too have been reported to enjoy the creative and informal approach to idea generation [20].

The use of cultural animation allowed people to connect with each other in a way that traditional methods do not [19, 21]. These connections were held in place by boundary objects, such as ribbons, buttons, fabric and picture frames, which symbolised a shared meaning between people and an exchange of knowledge and emotion. Asking groups to create an art installation using these objects further fostered teamwork and collaboration, both at an individual and collective level. The exploration of a creative self increased energy levels and encouraged productive discussions and problem-solving [19]. Objects also encouraged a solution-focused approach and permitted people to think beyond their usual everyday scope [17]. They also allowed facilitators to probe deeper about the greater meanings carried by the object, which acted as a metaphor [21].

From the researcher’s point of view, co-creative methods gave rise to ideas they might not have initially considered. Valaitis et al. [22], found that over 40% of the creative outputs were novel ideas brought to light by patients, healthcare providers/community care providers, community service providers and volunteers. One researcher commented, “It [the creative methods] took me on a journey, in a way that when we do other pieces of research it can feel disconnected” [23]. Another researcher also stated they could not return to the way they used to do research, as they have learnt so much about their own health and community and how they are perceived [19]. This demonstrates that creative processes not only benefit the project outcomes and the PPI group, but also facilitators and researchers. However, although engaging, creative methods have been criticised for not demonstrating academic rigour [17]. Moreover, creative PPI may also be exclusive to people who do not like or enjoy creative activities.

#### Creative PPI methods are cost and time efficient

Creative PPI workshops can often produce output that is visible and tangible. This can save time and money in the long run as the output is either ready to be implemented in a healthcare setting or a first iteration has already been developed. This may also offset the time and costs it takes to implement creative PPI. For example, the prototype of the decision support tool for people with malignant pleural effusion was developed using personas and creative worksheets. The end result was two tangible prototypes to drive the initial idea forward as something to be used in practice [17]. The use of creative co-design in this case saved clinician time as well as the time it would take to develop this product without the help of its end-users. In the development of this particular prototype, analysis was iterative and informed the next stage of development, which again saved time. The same applies for the feedback questionnaire for the assessment of ICT delivered aphasia rehabilitation. The co-created questionnaire, designed with people with aphasia, was ready to be used in practice [18]. This suggests that to overcome time and resource barriers to creative PPI, researchers should aim for it to be engaging whilst also producing output.

That useable products are generated during creative workshops signals to participating patients and public members that they have been listened to and their thoughts and opinions acted upon [23]. For example, the development of the back pain resource based on patient experiences implies that their suggestions were valid and valuable. Further, those who participated in the cultural animation workshop reported that the process visualises change, and that it already feels as though the process of change has started [19].

The most cost and time efficient method of creative PPI in this review is most likely the use of Facebook to gather feedback on project methodology [15]. Although there were drawbacks to this, researchers could involve more people from a range of geographical areas at little to no cost. Feedback was instantaneous and no training was required. From the perspective of the PPI group, they could interact however much or little they wish with no time commitment.

## Discussion

This systematic review identified four limitations and five strengths to the use of creative PPI in health and social care research. Creative PPI is time and resource intensive, can raise ethical issues and lacks generalisability. It is also not accepted by the mainstream. These factors may act as barriers to the implementation of creative PPI. However, creative PPI disrupts traditional power hierarchies and creates a safe space for taboo or mundane topics. It is also engaging, inclusive and can be time and cost efficient in the long term.

Something that became apparent during data analysis was that these are not blanket strengths and limitations of creative PPI as a whole. The umbrella term ‘creative PPI’ is broad and encapsulates a wide range of activities, ranging from music and poems to prototype development and persona-scenarios, to more simplistic things like the use of sticky notes and ordering cards. Many different activities can be deemed ‘creative’ and the strengths and limitations of one does not necessarily apply to another. For example, cultural animation takes greater effort to prepare than the use of sticky notes and sorting cards, and the use of Facebook is cheaper and wider reaching than persona development. Researchers should use their discretion and weigh up the benefits and drawbacks of each method to decide on a technique which suits the project. What might be a limitation to creative PPI in one project may not be in another. In some cases, creative PPI may not be suitable at all.

Furthermore, the choice of creative PPI method also depends on the needs and characteristics of the PPI group. Children, adults and people living with dementia or language difficulties all have different engagement needs and capabilities. This indicates that creative PPI is not one size fits all and that the most appropriate method will change depending on the composition of the group. The choice of method will also be determined by the constraints of the research project, namely time, money and the research aim. For example, if there are time constraints, then a method which yields a lot of data and requires a lot of preparation may not be appropriate. If generalisation is important, then an online method is more suitable. Together this indicates that the choice of creative PPI method is highly individualised and dependent on multiple factors.

Although the limitations discussed in this review apply to creative PPI, they are not exclusive to creative PPI. Ethical issues are a consideration within general PPI research, especially when working with more vulnerable populations, such as children or adults living with a disability. It can also be the case that traditional PPI methods lack generalisability, as people who volunteer to be part of such a group are more likely be older, middle class and retired [24]. Most research is vulnerable to this type of bias, however, it is worth noting that generalisation is not always a goal and research remains valid and meaningful in its absence. Although online methods may somewhat combat issues related to generalisability, these methods still exclude people who do not have access to the internet/technology or who choose not to use it, implying that online PPI methods may not be wholly representative of the general population. Saying this, however, the accessibility of creative PPI techniques differs from person to person, and for some, online mediums may be more accessible (for example for those with a physical disability), and for others, this might be face-to-face. To combat this, a range of methods should be implemented. Planning multiple focus group and interviews for traditional PPI is also time and resource intensive, however the extra resources required to make this creative may be even greater. Although, the rich data provided may be worth the preparation and analysis time, which is also likely to depend on the number of participants and workshop sessions required. PPI, not just creative PPI, often requires the provision of a financial incentive, refreshments, parking and accommodation, which increase costs. These, however, are imperative and non-negotiable, as they increase the accessibility of research, especially to minority and lower-income groups less likely to participate. Adequate funding is also important for co-design studies where repeated engagement is required. One barrier to implementation, which appears to be exclusive to creative methods, however, is that creative methods are not mainstream. This cannot be said for traditional PPI as this is often a mandatory part of research applications.

Regarding the strengths of creative PPI, it could be argued that most appear to be exclusive to creative methodologies. These are inclusive by nature as multiple approaches can be taken to evoke ideas from different populations - approaches that do not necessarily rely on verbal or written communication like interviews and focus groups do. Given the anonymity provided by some creative methods, such as personas, people may be more likely to discuss their personal experiences under the guise of a general end-user, which might be more difficult to maintain when an interviewer is asking an individual questions directly. Additionally, creative methods are by nature more engaging and interactive than traditional methods, although this is a blanket statement and there may be people who find the question-and-answer/group discussion format more engaging. Creative methods have also been cited to eliminate power imbalances which exist in traditional research [12, 13, 17, 19, 23]. These imbalances exist between researchers and policy makers and adolescents, adults and the community. Lastly, although this may occur to a greater extent in creative methods like prototype development, it could be suggested that PPI in general – regardless of whether it is creative - is more time and cost efficient in the long-term than not using any PPI to guide or refine the research process. It must be noted that these are observations based on the literature. To be certain these differences exist between creative and traditional methods of PPI, direct empirical evaluation of both should be conducted.

To the best of our knowledge, this is the first review to identify the strengths and limitations to creative PPI, however, similar literature has identified barriers and facilitators to PPI in general. In the context of clinical trials, recruitment difficulties were cited as a barrier, as well as finding public contributors who were free during work/school hours. Trial managers reported finding group dynamics difficult to manage and the academic environment also made some public contributors feel nervous and lacking confidence to speak. Facilitators, however, included the shared ownership of the research – something that has been identified in the current review too. In addition, planning and the provision of knowledge, information and communication were also identified as facilitators [25]. Other research on the barriers to meaningful PPI in trial oversight committees included trialist confusion or scepticism over the PPI role and the difficulties in finding PPI members who had a basic understanding of research [26]. However, it could be argued that this is not representative of the average patient or public member. The formality of oversight meetings and the technical language used also acted as a barrier, which may imply that the informal nature of creative methods and its lack of dependency on literacy skills could overcome this. Further, a review of 42 reviews on PPI in health and social care identified financial compensation, resources, training and general support as necessary to conduct PPI, much like in the current review where the resource intensiveness of creative PPI was identified as a limitation. However, others were identified too, such as recruitment and representativeness of public contributors [27]. Like in the current review, power imbalances were also noted, however this was included as both a barrier and facilitator. Collaboration seemed to diminish hierarchies but not always, as sometimes these imbalances remained between public contributors and healthcare staff, described as a ‘them and us’ culture [27]. Although these studies compliment the findings of the current review, a direct comparison cannot be made as they do not concern creative methods. However, it does suggest that some strengths and weaknesses are shared between creative and traditional methods of PPI.

### Strengths and limitations of this review

Although a general definition of creative PPI exists, it was up to our discretion to decide exactly which activities were deemed as such for this review. For example, we included sorting cards, the use of interactive whiteboards and sticky notes. Other researchers may have a more or less stringent criteria. However, two reviewers were involved in this decision which aids the reliability of the included articles. Further, it may be that some of the strengths and limitations cannot fully be attributed to the creative nature of the PPI process, but rather their co-created nature, however this is hard to disentangle as the included papers involved both these aspects.

During screening, it was difficult to decide whether the article was utilising creative qualitative methodology or creative *PPI*, as it was often not explicitly labelled as such. Regardless, both approaches involved the public/patients refining a healthcare product/service. This implies that if this review were to be replicated, others may do it differently. This may call for greater standardisation in the reporting of the public’s involvement in research. For example, the NIHR outlines different approaches to PPI, namely “consultation”, “collaboration”, “co-production” and “user-controlled”, which each signify an increased level of public power and influence [28]. Papers with elements of PPI could use these labels to clarify the extent of public involvement, or even explicitly state that there was no PPI. Further, given our decision to include only scholarly peer-reviewed literature, it is possible that data were missed within the grey literature. Similarly, the literature search will not have identified all papers relating to different types of accessible inclusion. However, the intent of the review was to focus solely on those within the definition of creative.

This review fills a gap in the literature and helps circulate and promote the concept of creative PPI. Each stage of this review, namely screening and quality appraisal, was conducted by two independent reviewers. However, four full texts could not be accessed during the full text reading stage, meaning there are missing data that could have altered or contributed to the findings of this review.

### Research recommendations

Given that creative PPI can require effort to prepare, perform and analyse, sufficient time and funding should be allocated in the research protocol to enable meaningful and continuous PPI. This is worthwhile as PPI can significantly change the research output so that it aligns closely with the needs of the group it is to benefit. Researchers should also consider prototype development as a creative PPI activity as this might reduce future time/resource constraints. Shifting from a top-down approach within research to a bottom-up can be advantageous to all stakeholders and can help move creative PPI towards the mainstream. This, however, is the collective responsibility of funding bodies, universities and researchers, as well as committees who approve research bids.

A few of the included studies used creative techniques alongside traditional methods, such as interviews, which could also be used as a hybrid method of PPI, perhaps by researchers who are unfamiliar with creative techniques or to those who wish to reap the benefits of both. Often the characteristics of the PPI group were not included, including age, gender and ethnicity. It would be useful to include such information to assess how representative the PPI group is of the population of interest.

## Conclusion

Creative PPI is a relatively novel approach of engaging the public and patients in research and it has both advantages and disadvantages compared to more traditional methods. There are many approaches to implementing creative PPI and the choice of technique will be unique to each piece of research and is reliant on several factors. These include the age and ability of the PPI group as well as the resource limitations of the project. Each method has benefits and drawbacks, which should be considered at the protocol-writing stage. However, given adequate funding, time and planning, creative PPI is a worthwhile and engaging method of generating ideas with end-users of research – ideas which may not be otherwise generated using traditional methods.

### Electronic supplementary material

Below is the link to the electronic supplementary material.

Additional file 1: Search strings: Description of data: the search strings and filters used in each of the 5 databases in this review

Additional file 2: Quality appraisal questions: Description of data: CASP quality appraisal questions

Additional file 3: Table 1: Description of data: elements of the data extraction table that are not in the main manuscript

## Data Availability

No datasets were generated or analysed during the current study.

## Abbreviations

CASP: Critical Appraisal Skills Programme
JBI: The Joanna Briggs Institute
NIHR: National Institute of Health and Care Research
PAG: Public Advisory Group
PPI: Public and Patient Involvement
WoS: Web of Science
