# Supplementary material for: What are the strengths and limitations to utilising creative methods in public and patient involvement in health and social care research? A qualitative systematic review
Source: Res Involv Engagem. 2024 May 13;10:48. doi: 10.1186/s40900-024-00580-4 (PMC11092192; doi:10.1186/s40900-024-00580-4)
Supplement: Supplementary file 1 — Additional file 1: Search strings: Description of data: the search strings and filters used in each of the 5 databases in this review [file 40900_2024_580_MOESM1_ESM.docx]

**Search strings**

**Web of Science**

Filtered by title and abstract.

Filtered 2009-2020, English language, human research.

AB=("creativ*" OR "innovat*" OR "authentic" OR "original" OR "inclu*") AND AB=("public and patient involvement" OR "patient and public involvement" OR "public and patient involvement and engagement" OR "patient and public involvement and engagement" OR "PPI" OR "PPIE" OR "co-produc*" OR "co-creat*" OR "co-design*" OR "cooperat*" OR "co-operat*") AND TI=("creativ*" OR "innovati*" OR "authentic" OR "original” OR “inclu*") AND TI=("public and patient involvement" OR "patient and public involvement" OR "public and patient involvement and engagement" OR "patient and public involvement and engagement" OR "PPI" OR "PPIE" OR "co-produc*" OR "co-creat*" OR "co-design*" OR "cooperat*" OR "co-operat*")

**Cochrane Library**

Filtered by title, abstract, keyword.

Filtered 2009 – 2023, English language, human research.

("creativ*" OR "innovat*" OR "authentic" OR "original" OR “inclu*”) AND ("public and patient involvement" OR "patient and public involvement" OR "public and patient involvement and engagement" OR "patient and public involvement and engagement" OR "PPI" OR "PPIE" OR "co-produc*" OR "co-creat*" OR "co-design*" OR "cooperat*" OR "co-operat*")

**PubMed**

Filtered by title

Filtered for 2009-2023, English language, human research.

(("creativ*"[Title] OR "innovat*"[Title] OR "authentic"[Title] OR "original"[Title] OR "inclu*"[Title])) AND (("public and patient involvement"[Title] OR "patient and public involvement"[Title] OR "public and patient involvement and engagement"[Title] OR "patient and public involvement and engagement"[Title] OR "PPI"[Title] OR "PPIE"[Title] OR "co-produc*"[Title] OR "co-creat*"[Title] OR "co-design*"[Title] OR "cooperat*"[Title] OR "co-operat*"[Title]))

**ASSIA**

Filtered by abstract

Filtered 2009-2023, English language, human research.

abstract(("creativ*" OR "innovat*" OR "authentic" OR "original" OR "inclu*")) AND abstract(("public and patient involvement" OR "patient and public involvement" OR "public and patient involvement and engagement" OR "patient and public involvement and engagement" OR "PPI" OR "PPIE" OR "co-produc*" OR "co-creat*" OR "co-design*" OR "cooperat*" OR "co-operat*"))

**CINAHL**

Filtered by title and abstract,

Filtered 2009-2023, English language, human research.

TI ( "creativ*" OR "innovat*" OR "authentic" OR "original” OR “inclu*" ) AND TI ( ("public and patient involvement" OR "patient and public involvement" OR "public and patient involvement and engagement" OR "patient and public involvement and engagement" OR "PPI" OR "PPIE" OR "co-produc*" OR "co-creat*" OR "co-design*" OR "cooperat*" OR "co-operat*" )

AB ( "creativ*" OR "innovat*" OR "authentic" OR "original” OR “inclu*" ) AND AB ( ("public and patient involvement" OR "patient and public involvement" OR "public and patient involvement and engagement" OR "patient and public involvement and engagement" OR "PPI" OR "PPIE" OR "co-produc*" OR "co-creat*" OR "co-design*" OR "cooperat*" OR "co-operat*" )
