# Supplementary material for: What are the strengths and limitations to utilising creative methods in public and patient involvement in health and social care research? A qualitative systematic review
Source: Res Involv Engagem. 2024 May 13;10:48. doi: 10.1186/s40900-024-00580-4 (PMC11092192; doi:10.1186/s40900-024-00580-4)
Supplement: Supplementary file 2 — Additional file 2: Quality appraisal questions: Description of data: CASP quality appraisal questions [file 40900_2024_580_MOESM2_ESM.docx]

**Quality appraisal questions**

**Answered Yes (Y), No (N) or Unknown (U).**

1. Was there a clear statement of the aims of the research?

2. Is a qualitative methodology appropriate?

3. Was the research design appropriate to address the aims of the research?

4. Was the recruitment strategy appropriate to the aims of the research?

5. Was the data collected in a way that addressed the research issue?

6. Has the relationship between researcher and participants been adequately considered?

7. Have ethical issues been taken into consideration?

8. Was the data analysis sufficiently rigorous?

9. Is there a clear statement of findings?

10. Is the research particularly valuable?
