# Supplementary material for: What are the strengths and limitations to utilising creative methods in public and patient involvement in health and social care research? A qualitative systematic review
Source: Res Involv Engagem. 2024 May 13;10:48. doi: 10.1186/s40900-024-00580-4 (PMC11092192; doi:10.1186/s40900-024-00580-4)
Supplement: Supplementary file 3 — Additional file 3: Table 1: Description of data: elements of the data extraction table that are not in the main manuscript [file 40900_2024_580_MOESM3_ESM.docx]

| Grindell et al | Galler et al | Fedorowicz et al | Craven et al | Cook et al | Byrne et al | **Author** |
| --- | --- | --- | --- | --- | --- | --- |
| Using creative co-design to develop a decision support tool for people with malignant pleural effusion. | Listening to children voices in early stages of new product development through co-creation-Creative focus group and online platform | Using social media for patient and public involvement and engagement in health research: The process and impact of a closed Facebook group | Try to see it my way: exploring the co-design of visual presentations of wellbeing through a workshop process. | Can you design the perfect condom? Engaging young people to inform safe sexual health practice and innovation. | The creative turn in evidence for public health: community and arts-based methodologies. | **Title** |
| To understand the lived experience of malignant pleural effusion and its management from both service user and provider perspectives to gain a shared understanding of the key issues to be addressed. | To assess a methodology for early-stage idea generation through co-creation, for the development of healthy snacks with pre-adolescents, and to compare two settings, creative focus groups (CFG) and an online community (ONL). | To explore the process and impact of conducting PPIE through a closed Facebook group | To develop the specification of a visualisation toolbox that could be applied on digital platforms (web or app-based) to support adults with lived experience of mental health difficulties to present and track their personal wellbeing in a multi-media format. | To destigmatise discussions about sexuality and contraception and to provide information about what characteristics a perfect condom might embody for adolescents. | To establish whether arts-based methodologies can be of value in the production and exchange of evidence in supporting public health related policy, particularly in the context of Welsh policy | **Aim** |
| 41 (made up of patients, carers, physicians, nurses) | 3 groups of 7 children (21) for creative workshops. 52 children for online platform | 289 | Workshop 1 = 13, workshop = 12 | Not specified | 15-200 | **Number of PPI contributors** |
| Does not produce generalizable solutions. Does not fulfil needs of academic rigour. Creative co-production is resource intensive. | Online methods required access to an electronical device and knowledge of how to operate it. | Exclusion of those without reliable internet access or who are unfamiliar with social media. Maintaining the Facebook group was a challenge for researchers; researchers engaged in discussion with group outside usual working hours. | The visualisations were complex and highly personal; showed idiosyncrasy. | Not specified | Arts-based methods present risk of over-disclosure and emotional distress. Loss of anonymity if participants want to be named for their creative work. | **Limitations** |
| The process overcame power, language and time issues… enabled people to share and express themselves in an inclusive environment using a common language. Gave participants the permission to think beyond the usual constraints of their working environment. Prototype development saved patient and clinician time. Prototyping turned participants’ ideas into something tangible that helped generate more useful and practical feedback to drive the idea forward. Increased sense of ownership. Co-design blurred traditional academic and practice boundaries. | Creative methods were highly engaging for participants and the creative focus group facilitated teamwork and group learning. | Offers flexibility that can be beneficial for both research teams and patients and the public. Allows for wider participation. Requires no training. Inclusive. Easier and less effort to provide feedback. No costs. Rapid feedback. Using a Facebook group allowed the researchers to seek feedback in a variety of ways from the group, such as comments, likes and voting in polls. These benefits have the potential to open PPIE up to a different cohort of people who previously would have found it difficult to commit to face-to-face meetings. | Created a safe peer group setting that invited participants to share openly. | Reduction in power imbalances between researchers and adolescents. The hypothetical nature allowed people to freely discuss experiences. This permitted an expanded set of ideas to be generated. Discussion without fear of discrimination or judgement. Produces more colourful data than traditional methods. | Can engage with decision-makers. Disrupts power - creates environment of understanding and respect. People communicate what might be 'unsayable' in terms of everyday, mundane experiences. | **Strengths** |
| Preferred methods for receiving information were varied but visual and graphic approaches were favoured. | Preadolescents can co-create food product ideas that could be applied in the early development stages of innovative healthy food and meals. | The use of a closed Facebook group can facilitate effective PPIE. Its flexibility can be beneficial for researchers, patients and public who wish to engage in the research process. Dedicated time for sustained group engagement is important. | Successful implementation of dynamic visualisation could enable predictive analysis of sustained wellbeing and potential relapse, facilitating lean consumption of scarce health and care resources. | It is possible to have rich conversations with young people about condoms and sexual health. Expression through art allows them to think about the condom in ways that extend beyond risk, prevention, and danger. Their insights can aid in the development of condoms that address the wants and needs of the adolescent community. | Arts-based methods can facilitate knowledge exchange between the public and decision makers. | **Main findings** |

Table 1 continued…

| Webber et al | Valaitis et al | Micsinszki et al | Keogh et al | Kelemen et al | Kearns et al | **Author** |
| --- | --- | --- | --- | --- | --- | --- |
| The creative co-design of low back pain education resources | Health TAPESTRY: co-designing interprofessional primary care programs for older adults using the persona-scenario method. | Creative processes in co-designing a co-design hub: towards system change in health and social services in collaboration with structurally vulnerable populations. | Innovative methods for involving people with dementia and carers in the policymaking process. | Cultural animation in health research: An innovative methodology for patient and public involvement and engagement | Rating experience of ICT-delivered aphasia rehabilitation: co-design of a feedback questionnaire. | **Title** |
| To use creative co-design methods to develop prototype back pain educational resources for use withing an NHS community physiotherapy service. | To report on how the persona-scenario method was used to co-design a complex primary health care intervention (Health TAPESTRY) by and for older adults and providers and the value added of this approach. | To create a Co-Design Hub for the health and well-being of vulnerable populations. This is to promote engagement, education and innovation to advance co-design in health and social services and research to address inequities. | To create a pathway for the voice and experiences of people with dementia and family carers to influence upcoming legislation on home care— currently represented through advocacy but not through direct voice. | With residents described by official documents as “leading unhealthy lifestyles”, this project aimed to explore the responses of community members to claims such as these to identify what “good health” meant for them, and finally what participants might do in response to this situation at a community and individual level. | To develop a questionnaire to facilitate feedback on ICT-delivered aphasia rehab by collaboratively working with people with aphasia in the design process, to describe the development process and the co-design techniques employed and to explore the experiences of co-designers in the development process. | **Aim** |
| 16 (8 public participants, 7 HCPs and 1 clinician, previously a GP). Not all participants attended both workshops. | Patients (n = 15), healthcare providers/community care providers (n = 29), community service providers (n = 12), and volunteers (n = 14). | 8 in café 1 and 12 in café 2 | 10 individuals with young onset and later onset dementia. 28 dementia caregivers | 20-25 | 6 people with mild-severe aphasia | **Number of PPI contributors** |
| Not specified | Funding for financial incentives e.g. Parking, food, transport.  Skilled facilitators need qualitative skills to probe for rich data The large volume of information produced was difficult to disseminate to scientific leads and was unwieldy to review in team meetings. Time required for analysis. | Lack of infrastructure to conduct meaningful co-creation, dominance of the medical model, exclusive procedures of healthcare organisations and professional regulators, environments that privilege individualism over collectivism, self-sufficiency over collaboration, and scientific expertise over other ways of knowing based on lived experiences. Financial support (accommodation/compensation). | PPI is tokenistic. Timing issues not allowing as much time as people would want for discussion. | Participatory arts-based methodologies are yet to become mainstream. | Time and labour intensive, especially to make aphasia accessible materials. | **Limitations** |
| Researchers feel more connected to the project. Output is contextually specific, evidence based. It encouraged collaborative problem solving and dissolved hierarchies. Useable product generated - demonstrates that what people have said have been listened to and acted upon. Increased ownership of project. Creative approaches allows people to externalise thoughts, feelings and experiences in a safe way | Utilising this approach allowed the intervention design to more fully capture each type of perspective and anticipate potential implementation challenges. The persona scenario method can inform the development of an intervention involving its end users as well as those delivering the intervention. Ideas generated that the research team may not originally think of. Participants enjoyed the process. | Boundary objects opens the conversation to allow the facilitator to probe deeper about what was meant by the metaphors they provide. Use of Google Jamboard allowed people to engage in real time during COVID. Graphic designer captured additional "movement and fluidity" and "people and relationships” - what might have been difficult to put into words. Inclusive, promoting and supporting plain, culturally appropriate language | Enjoyment of creative elements and informal approach. Useful method for people with communication difficulties. | CA dissolves hierarchies and traditional barriers associated with professional expertise. Everyone’s contribution equally important. Fosters human connectivity using boundary objects. Allows people to find and express a creative self, which lead to increased levels of energy, productive discussions and problem solving. People from different backgrounds can connect, communicate and achieve consensus. People not overwhelmed by words, methodology visualises change, people felt like they had already started the process of change. The process was described as democratic, empowering, honest, practical and valuable.  CA can ensure that the reporting of the research findings, which remains problematic in PPIE research, can take place both via conventional scholarly outlets, such as conference presentations, journal papers and academic books, and in venues where the wider public can more easily engage with the findings (public exhibitions, performances, podcasts and blogs). | Accessible. Inclusive. Co-designers reported feeling comfortable about contributing in workshops. Brings people together to talk and relate, supposed to individual interviews. Iterative design process | **Strengths** |
| To bring about meaningful change, evidence-based guidelines need to be implemented in ways that are sensitive to context and the complexity of healthcare. Co-production has the potential to produce better solutions but has its own challenges. Creative co-design can be an effective approach for overcoming these challenges. | The persona-scenario method drew out feasible novel ideas from stakeholders, which expanded on the research team's original ideas and highlighted interactions among components and stakeholder groups. Many ideas were integrated into the Health TAPESTRY program's design and implementation. | Co-design processes are complex, requiring vulnerability, trust, flexibility, and a willingness to create and sustain change. Innovative co-creation methods can elicit diverse experiences and impact change in services, systems, and policies. | Involving people with dementia and carers in policy development requires time and creativity to facilitate and maximize their involvement. Co-production is essential to ensure the priorities of participants are identified, expressed and communicated effectively. | CA provides a route to co-produce research agendas, empowers the public to engage actively with health professionals and make a positive contribution to their community | The co-design process provided opportunities for social interaction with other people with aphasia and allowed co-designers to reflect on their own abilities. | **Main findings** |
